# Association between maternal *MTHFR* C677T/A1298C combination polymorphisms and IVF/ICSI outcomes: a retrospective cohort study

**DOI:** 10.1093/hropen/hoac055

**Published:** 2022-12-05

**Authors:** Yong-Jie Lu, Qin Li, Li-Xue Chen, Tian Tian, Jia Kang, Yong-Xiu Hao, Jian-Suo Zhou, Yuan-Yuan Wang, Li-Ying Yan, Rong Li, Liang Chang, Jie Qiao

**Affiliations:** Center for Reproductive Medicine, Department of Obstetrics and Gynecology, Peking University Third Hospital, Beijing, China; National Clinical Research Center for Obstetrics and Gynecology, Peking University Third Hospital, Beijing, China; Key Laboratory of Assisted Reproduction, Peking University, Ministry of Education, Beijing, China; Beijing Key Laboratory of Reproductive Endocrinology and Assisted Reproductive Technology, Beijing, China; Center for Reproductive Medicine, Department of Obstetrics and Gynecology, Peking University Third Hospital, Beijing, China; National Clinical Research Center for Obstetrics and Gynecology, Peking University Third Hospital, Beijing, China; Key Laboratory of Assisted Reproduction, Peking University, Ministry of Education, Beijing, China; Beijing Key Laboratory of Reproductive Endocrinology and Assisted Reproductive Technology, Beijing, China; Center for Reproductive Medicine, Department of Obstetrics and Gynecology, Peking University Third Hospital, Beijing, China; National Clinical Research Center for Obstetrics and Gynecology, Peking University Third Hospital, Beijing, China; Key Laboratory of Assisted Reproduction, Peking University, Ministry of Education, Beijing, China; Beijing Key Laboratory of Reproductive Endocrinology and Assisted Reproductive Technology, Beijing, China; Center for Reproductive Medicine, Department of Obstetrics and Gynecology, Peking University Third Hospital, Beijing, China; National Clinical Research Center for Obstetrics and Gynecology, Peking University Third Hospital, Beijing, China; Key Laboratory of Assisted Reproduction, Peking University, Ministry of Education, Beijing, China; Beijing Key Laboratory of Reproductive Endocrinology and Assisted Reproductive Technology, Beijing, China; Center for Reproductive Medicine, Department of Obstetrics and Gynecology, Peking University Third Hospital, Beijing, China; National Clinical Research Center for Obstetrics and Gynecology, Peking University Third Hospital, Beijing, China; Key Laboratory of Assisted Reproduction, Peking University, Ministry of Education, Beijing, China; Beijing Key Laboratory of Reproductive Endocrinology and Assisted Reproductive Technology, Beijing, China; Center for Reproductive Medicine, Department of Obstetrics and Gynecology, Peking University Third Hospital, Beijing, China; National Clinical Research Center for Obstetrics and Gynecology, Peking University Third Hospital, Beijing, China; Key Laboratory of Assisted Reproduction, Peking University, Ministry of Education, Beijing, China; Beijing Key Laboratory of Reproductive Endocrinology and Assisted Reproductive Technology, Beijing, China; Department of Laboratory Medicine, Peking University Third Hospital, Beijing, China; Center for Reproductive Medicine, Department of Obstetrics and Gynecology, Peking University Third Hospital, Beijing, China; National Clinical Research Center for Obstetrics and Gynecology, Peking University Third Hospital, Beijing, China; Key Laboratory of Assisted Reproduction, Peking University, Ministry of Education, Beijing, China; Beijing Key Laboratory of Reproductive Endocrinology and Assisted Reproductive Technology, Beijing, China; Center for Reproductive Medicine, Department of Obstetrics and Gynecology, Peking University Third Hospital, Beijing, China; National Clinical Research Center for Obstetrics and Gynecology, Peking University Third Hospital, Beijing, China; Key Laboratory of Assisted Reproduction, Peking University, Ministry of Education, Beijing, China; Beijing Key Laboratory of Reproductive Endocrinology and Assisted Reproductive Technology, Beijing, China; Center for Reproductive Medicine, Department of Obstetrics and Gynecology, Peking University Third Hospital, Beijing, China; National Clinical Research Center for Obstetrics and Gynecology, Peking University Third Hospital, Beijing, China; Key Laboratory of Assisted Reproduction, Peking University, Ministry of Education, Beijing, China; Beijing Key Laboratory of Reproductive Endocrinology and Assisted Reproductive Technology, Beijing, China; Center for Reproductive Medicine, Department of Obstetrics and Gynecology, Peking University Third Hospital, Beijing, China; National Clinical Research Center for Obstetrics and Gynecology, Peking University Third Hospital, Beijing, China; Key Laboratory of Assisted Reproduction, Peking University, Ministry of Education, Beijing, China; Beijing Key Laboratory of Reproductive Endocrinology and Assisted Reproductive Technology, Beijing, China; Center for Reproductive Medicine, Department of Obstetrics and Gynecology, Peking University Third Hospital, Beijing, China; National Clinical Research Center for Obstetrics and Gynecology, Peking University Third Hospital, Beijing, China; Key Laboratory of Assisted Reproduction, Peking University, Ministry of Education, Beijing, China; Beijing Key Laboratory of Reproductive Endocrinology and Assisted Reproductive Technology, Beijing, China

**Keywords:** MTHFR, gene polymorphism, assisted reproductive technology, oocyte maturation, embryo quality

## Abstract

**STUDY QUESTION:**

What are the roles of maternal 5,10-methylenetetrahydrofolate reductase (*MTHFR*) C677T/A1298C combination polymorphisms on the embryological and clinical outcomes of IVF/ICSI?

**SUMMARY ANSWER:**

Our study reveals for the first time that the oocyte maturation potential gradually decreases with a reduction of maternal MTHFR activity determined by combined C677T/A1298C polymorphisms, while embryo quality was worse in women with intermediate MTHFR activity.

**WHAT IS KNOWN ALREADY:**

Although many previous studies have explored the association between *MTHFR* polymorphisms and IVF/ICSI outcomes, the results remain contradictory due to inadequate samples, no adjustment for potential confounders and/or the study of C677T and A1298C separately. Few studies have systematically investigated the exact role of MTHFR activity determined by combined C677T/A1298C polymorphisms on the embryological and clinical outcomes of IVF/ICSI.

**STUDY DESIGN, SIZE, DURATION:**

This is a retrospective cohort study investigating 1160 women who were referred for *MTHFR* genotyping and IVF/ICSI treatment at Peking University Third Hospital from May 2017 to May 2020.

**PARTICIPANTS/MATERIALS, SETTING, METHODS:**

Women who were referred for *MTHFR* genotyping and their first IVF/ICSI treatment at our hospital were included and those undergoing preimplantation genetic testing cycles were excluded. The included women were divided into different cohorts according to their C677T, A1298C and combined C677T/A1298C genotypes. The embryological outcomes, including oocytes retrieved, metaphase II oocytes, oocyte maturation rate, normal fertilization rate and transplantable embryo rate, were evaluated by generalized linear regression models. The clinical outcomes, including biochemical pregnancy rate, clinical pregnancy rate and live birth rate, were evaluated by log-binomial regression models. All outcomes were adjusted for potential confounders.

**MAIN RESULTS AND THE ROLE OF CHANCE:**

Women with the combined 677TT/1298AA genotype (hereafter abbreviated as TT/AA, as with other combined genotypes), whose enzyme activity was the lowest, had a lower oocyte maturation rate compared with those with the wild-type genotype (*P *=* *0.007). Moreover, the oocyte maturation rate decreased linearly with the decline in MTHFR enzyme activity determined by combined C677T/A1298C genotypes (*P*-trend = 0.001). The combined CC/AC, CC/CC&CT/AA and CT/AC genotypes with intermediate enzyme activity were associated with a lower transplantable embryo rate (*P *=* *0.013, 0.030 and 0.039, respectively). The differences in clinical outcomes between women with wild-type genotype and combined C677T/A1298C variant genotypes were not significant.

**LIMITATIONS, REASONS FOR CAUTION:**

Our study population had comparable embryological outcomes but worse clinical outcomes than other women undergoing IVF/ICSI treatment at our hospital. Therefore, the results related to the clinical outcomes should be generalized with caution. In addition, we did not detect the folate concentration of each patient during pregnancy. However, this might not have much influence on our results because almost all of our study participants took sufficient folic acid around pregnancy.

**WIDER IMPLICATIONS OF THE FINDINGS:**

We provide a holistic view of the effect of *MTHFR* C677T and A1298C polymorphisms on the IVF/ICSI outcomes, which can contribute to providing reasonable folic acid supplementation suggestions for women with different *MTHFR* genotypes, especially for those with a low oocyte maturation rate and/or low embryo quality.

**STUDY FUNDING/COMPETING INTERESTS:**

This work was funded by the National Natural Science Foundation of China (31871447, and 82101677), the National Key Research and Development Program (2019YFA0801400) and the Natural Science Foundation of Beijing Municipality (7202226). The authors declare that they have no competing interests.

**TRIAL REGISTRATION NUMBER:**

N/A.

WHAT DOES THIS MEAN FOR PATIENTS? This study focuses on how the two most common variations of the *MTHFR* gene jointly affect assisted reproductive treatment outcomes.

There is currently no reliable conclusion about the relationship between the two most common variations of *MTHFR* and the outcomes of assisted reproduction, and a lack of evidence on the combined effect of the two variations.

This study systematically compared assisted reproductive outcomes in women with different *MTHFR* combined genotypes and found that lower MTHFR protein activity, determined by the combined genotypes, was associated with lower oocyte maturation potential, and embryo quality was also associated with the combined genotypes.

The researchers believe that the effects of maternal *MHTFR* variations on oocytes/embryos may have an impact on the postnatal health of offspring or the health of grandoffspring, which is worthy of further research.

## Introduction

Infertility is a global health issue affecting ∼10–15% of couples worldwide (Datta *et al.*, 2016). Although ART has made significant progress in recent years, its success rate is still only approximately 30% per cycle (Kushnir *et al.*, 2017). Exploring the latent factors affecting ART outcomes is therefore of great significance for treating infertility.

Accumulating evidence indicates that folate metabolism has an impact on human reproductive health including ART outcomes (Forges *et al.*, 2007; Gernand *et al.*, 2016). Folate-mediated one-carbon metabolism is necessary for DNA, RNA and protein synthesis, for balancing the homocysteine level and for providing methyl donors for methylation (Ducker and Rabinowitz, 2017). Adequate folate is essential for follicular and embryonic development as cells proliferate rapidly during folliculogenesis and pregnancy (Lassi *et al.*, 2013). 5,10-methylenetetrahydrofolate reductase (MTHFR), which catalyzes the reduction of 5,10-methylenetetrahydrofolate into 5-methyltetrahydrofolate, is a crucial enzyme in folate metabolism; its polymorphisms can cause disturbances in folate and homocysteine status due to a reduction in the enzyme activity (Thomas and Fenech, 2008). C677T (rs1801133) and A1298C (rs1801131) are the two most common and well-described polymorphisms of *MTHFR*. The enzyme activity in the 677CT and 677TT genotypes is only 65% and 30% of that in the 677CC genotype, respectively (Frosst *et al.*, 1995), whereas the A1298C polymorphism is related to a slighter decrease in enzyme activity (Weisberg *et al.*, 1998).

Some studies have suggested that variation at the *MTHFR* 677 locus is associated with fewer oocytes retrieved (Thaler *et al.*, 2006; Pavlik *et al.*, 2011), a lower oocyte maturation rate (D'Elia *et al.*, 2014), a lower fertilization rate (Marci *et al.*, 2012), a higher miscarriage rate (Nair *et al.*, 2012; Xu *et al.*, 2019) or a higher risk of small for gestational age (Bulloch *et al.*, 2020), while other studies have not confirmed such associations (Dobson *et al.*, 2007; Rosen *et al.*, 2007; Marci *et al.*, 2012; D'Elia *et al.*, 2014; Aguilar-Lacasaña *et al.*, 2021; Wang *et al.*, 2021) or even yielded opposite results (Laanpere *et al.*, 2011). Also, previous studies have yielded conflicting results on the correlation between A1298C polymorphism and reproductive outcomes (Haggarty *et al.*, 2006; Dobson *et al.*, 2007; Rosen *et al.*, 2007; Laanpere *et al.*, 2011; D'Elia *et al.*, 2014; Xu *et al.*, 2019; Bulloch *et al.*, 2020; Wang *et al.*, 2021). Thereby, there is currently no consistent and convincing conclusion on the relationship between *MTHFR* polymorphisms and reproductive outcomes. This may be due to one or more of the following shortcomings that have existed in previous studies: (i) the sample sizes have been small, ranging from 60 to 1011 cases, (ii) some had no eligibility criteria (Dobson *et al.*, 2007; Rosen *et al.*, 2007; Laanpere *et al.*, 2011) or did not adjust for potential confounding factors (Marci *et al.*, 2012; Nair *et al.*, 2012; D'Elia *et al.*, 2014) and, most importantly, (iii) the vast majority of previous studies have investigated the relationship between C677 or A1298C polymorphism and reproductive outcomes separately. However, the two most common polymorphisms, C677T and A1298C, determine MTHFR activity jointly rather than independently. Therefore, the results from studying one of the two polymorphisms alone may be biased by differences in the distribution of the other polymorphism between groups.

Taking the above into consideration, we conducted this retrospective cohort study by recruiting up to 1160 women who were referred for *MTHFR* genotyping and IVF/ICSI treatment, while adjusting for potential confounders, and analyzing C677T and A1298C polymorphisms in combination, to provide convincing evidence of the relationship between *MTHFR* C677T/A1298C combination polymorphisms and IVF/ICSI outcomes.

## Materials and methods

### Study participants

**Figure 1.**
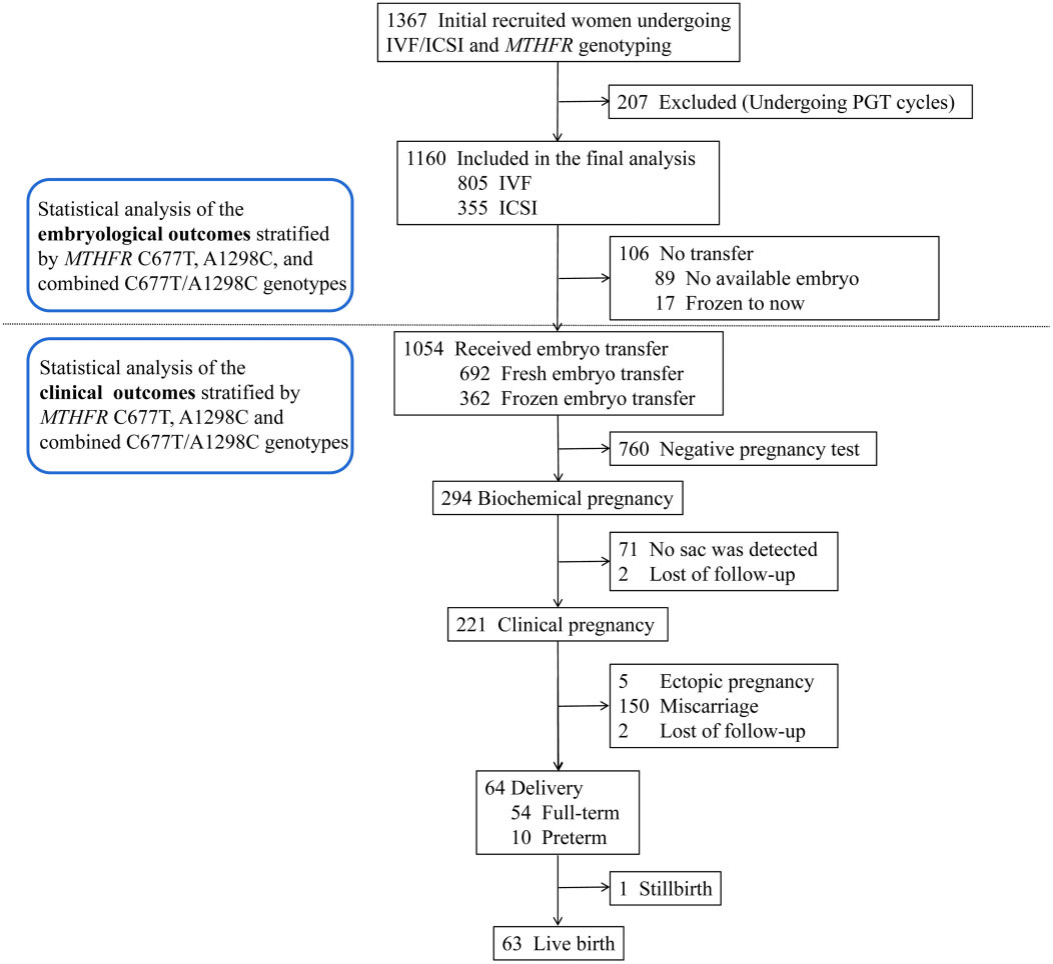
**The study flow chart.** The detailed inclusion/exclusion process and the number of women at each stage.

This is a retrospective cohort study. Infertile women who were referred to the Center for Reproductive Medicine of Peking University Third Hospital for *MTHFR* genotyping and IVF/ICSI treatment from May 2017 to May 2020 were included. For each woman, only the first ovarian stimulation cycle and its first corresponding embryo transfer or frozen-thawed embryo transfer cycle during this period were included. Participants who underwent a preimplantation genetic testing cycle due to a high risk of genetic variation in their gametes, such as those who (or their spouses) carried a definite chromosome abnormality (translocation, inversion, etc.) or a gene mutation, were excluded because the high proportion of gametes with chromosomal abnormalities and gene mutations, as well as the genetic detection and artificial selection of embryos for these participants, would significantly bias the embryological and clinical outcomes. Finally, a total of 1160 women were included in subsequent analyses (Fig. 1). Follow-up was performed at 2 weeks, 3 weeks, 4–6 weeks after embryo transfer and 1 and 2 weeks after delivery by telephone or outpatient review. All participants were advised to take 800 μg of folic acid daily for 3 months before and after pregnancy.

### Ethical approval

This study was approved by the Peking University Third Hospital Medical Science Research Ethics Committee (IRB00006761-M2020004) and informed consent was obtained from all participants.

### Genotyping

The *MTHFR* genotyping results were collected from medical records. The genotyping was performed as follows. The peripheral blood was collected, and genomic DNA was extracted using the TIANamp Blood DNA Kit (Tiangen Biotech Co., Ltd., Beijing, China) according to the manufacturer’s instructions. The *MTHFR* C677T and A1298C genotypes were determined by performing a fluorescence quantitative PCR using an *MTHFR* genotype identification kit (NuoDe Medical Co., Ltd., Nanchang, China). The premix in the kit contained all components except for the DNA template. The primers and probes were as follows: *MTHFR* C677T forward primer 5′-CCGAAGCAGGGAGCTTTG-3′, reverse primer 5′-CGGTGCATGCCTTCACAA-3′, probe 1 (VIC-labeled) AAATCG[G]CTCCCGC, probe 2 (FAM-labeled) AAATCG[A]CTCCCGC; *MTHFR* A1298C forward primer 5′-CCCGAGAGGTAAAGAACGAAGAC-3′, reverse primer 5′-AGGAGGAGCTGCTGAAGATGTG-3′, probe 1 (VIC-labeled) AAAGACACTT[G]CTTCACTGG and probe 2 (FAM-labeled) AAAGACACTT[T]CTTCACTGG. Fluorescence quantitative PCR was performed on a Cobas Z480 Cycler (Roche Diagnostics, Switzerland), and the reaction system contained 1 μl of 20 ng/μl DNA and 19 μl of premix. The PCR amplification conditions were 95°C for 10 min, then 40 cycles of 95°C for 15 s and 60°C for 40 s and, finally, the reaction was maintained at 4°C. Data were analyzed with LightCycler^®^480 SW 1.5 software (Roche, Switzerland).

### Variables and assessment of outcomes

The following variables were included for the statistical analysis: maternal age, maternal BMI, paternal age, paternal BMI, maternal cause of infertility, paternal cause of infertility, infertility type, stimulation protocol, fertilization type, embryo transfer method and type of embryos transferred.

The embryological outcomes were assessed as follows. After receiving the stimulation protocol according to the condition of each patient, the oocytes were retrieved 36 hours after the hCG trigger. Mature oocytes (metaphase II [MII] oocytes) were defined as the presence of a first polar body (PB) and subsequently used for ICSI. The oocyte maturation rate was defined as the ratio of MII oocytes to the number of oocytes retrieved. Normal fertilization was confirmed by the presence of two pronuclei (PN) and a second PB 16–18 hr after insemination, and the normal fertilization rate was obtained by dividing the number of oocytes with normal fertilization by the number of oocytes inseminated. At 67–69 hr (Day 3) after insemination, the embryo quality was assessed according to the cell numbers and the degree of cytoplasmic fragmentation. The transplantable embryo rate was calculated by dividing the number of embryos that developed from 2PN and reached five or more cell stages with less than 30% fragmentation on Day 3 by the number of cleaved embryos on Day 2. Ultimately, Day 3, Day 5 or Day 6 embryos were transferred according to each patient’s condition.

Regarding the clinical outcomes, biochemical pregnancy was defined as serum β-hCG level >10 IU/l measured 14 days after embryo transfer. Clinical pregnancy was defined as ultrasonographic visualization of at least one gestational sac 30 days after embryo transfer. Live birth was defined as the birth of one or more live infants.

### Statistical analysis

Categorical variables were reported as number (percentage) and compared by the Chi-square test or Fisher’s exact test. Continuous variables that were not normally distributed were presented as median (25th, 75th percentile) and compared by Mann–Whitney *U* test. The Hardy–Weinberg equilibrium test was used to evaluate the genetic equilibrium of the included participants. Generalized linear regression models with the adjustment for potential confounders were applied to evaluate the associations between *MTHFR* genotypes and the embryological outcomes of IVF/ICSI. Adjusted means, coefficients, 95% CI, *P*-values for the difference and *P*-values for linear trend were calculated. Adjusted relative risks (aRR) and 95% CI derived from log-binomial regression models were used to compare the biochemical pregnancy rate, clinical pregnancy rate and live birth rate between different genotypes. Potential confounders were selected on the basis of clinical knowledge and were listed in the corresponding table legends. SPSS version 20.0 (IBM, Inc.) software was used for the statistical analysis. *P* < 0.05 was considered statistically significant.

## Results

### Baseline characteristics

Figure 1 shows the detailed inclusion/exclusion process and the number of women at each stage. As shown in Table I, the final analytical sample comprised 1160 women, including 123 (10.6%) women with the 677CC/1298AA genotype (abbreviated as CC/AA, so were other combined genotypes) and 97 (8.4%), 395 (34.1%), 180 (15.5%) and 365 (31.5%) women with combined variant genotypes CC/AC, CC/CC&CT/AA, CT/AC and TT/AA, respectively. Three-variation genotypes (TT/AC and CT/CC) and four-variation genotype (TT/CC), which were extremely rare in adults probably due to their non-viability for individuals (Callejón *et al.*, 2007), were not present in our study population. *MTHFR* genotypes were listed in the descending order of their corresponding enzyme activities from left to right in the table. The enzyme activity determined *in vitro* and *in vivo* was CC/AA > CC/AC > CC/CC&CT/AA > CT/AC > TT/AA (van der Put *et al.*, 1998; Weisberg *et al.*, 1998; Ulvik *et al.*, 2007). The CC/CC and CT/AA genotypes were merged into one group named ‘CC/CC&CT/AA’ due to their comparable enzyme activity. Our further analysis of the linear relationship between homocysteine concentration and the activity of MTHFR enzyme in 3923 women who visited our hospital reconfirmed this order *in vivo* (Supplementary Table SI). The frequencies of *MTHFR* genotypes were 21.3% (n = 247) for 677CC, 47.2% (n = 548) for 677CT, 31.5% (n = 365) for 677TT, 73.8% (n = 856) for 1298AA, 23.9% (n = 277) for 1298AC and 2.3% (n = 27) for 1298CC in our study population, which showed a comparable distribution pattern with the general Han women in northern China (Wang *et al.*, 2016) (Supplementary Table SII). Both C677T and A1289C genotypes were in Hardy–Weinberg equilibrium (Supplementary Table SIII).

**Table I:** Baseline characteristics of participants with different *MTHFR* C677T/A1298C combination genotypes.

Characteristic	All participants	CC/AA	CC/AC	CC/CC&CT/AA	CT/AC	TT/AA	*P*-value
**n (%)**	1160 (100.0)	123 (10.6)	97 (8.4)	395 (34.1)	180 (15.5)	365 (31.5)	
**Maternal age, years**	33.0 (31.0, 36.0)	33.0 (31.0, 36.0)	34.0 (32.0, 37.0)	33.0 (31.0, 36.0)	33.0 (30.5, 36.0)	33.0 (30.0, 36.0)	0.051
**Paternal age, years**	34.0 (31.0, 38.0)	34.0 (31.0, 36.5)	35.0 (32.0, 38.0)	34.0 (31.0, 37.0)	34.0 (31.0, 38.0)	34.0 (30.0, 38.0)	0.208
**Maternal BMI, kg/m^2^**	22.0 (20.2, 24.5)	22.0 (20.1, 24.1)	21.8 (20.2, 23.9)	21.5 (20.0, 23.8)	22.3 (20.7, 25.4)	22.1 (20.3, 25.4)	**0.020**
**Paternal BMI, kg/m^2^**	25.5 (23.4, 27.8)	25.0 (23.1, 26.2)	25.8 (23.7, 27.7)	25.7 (23.5, 27.9)	25.6 (22.9, 28.4)	25.5 (23.4, 27.5)	0.133
**Maternal cause of infertility**							0.356
Unexplained	349 (30.1)	30 (24.4)	38 (39.2)	122 (30.9)	51 (28.3)	108 (29.6)	
Tubal factor	370 (31.9)	45 (36.6)	33 (34.0)	114 (28.9)	65 (36.1)	113 (31.0)	
PCOS	138 (11.9)	13 (10.6)	6 (6.2)	47 (11.9)	24 (13.3)	48 (13.2)	
Diminished ovarian reserve	149 (12.8)	18 (14.6)	12 (12.4)	56 (14.2)	18 (10.0)	45 (12.3)	
Endometriosis	54 (4.7)	8 (6.5)	6 (6.2)	19 (4.8)	7 (3.9)	14 (3.8)	
Other	100 (8.6)	9 (7.3)	2 (2.1)	37 (9.4)	15 (8.3)	37 (10.1)	
**Paternal cause of infertility**							0.479
Unexplained	576 (49.7)	69 (56.1)	52 (53.6)	193 (48.9)	89 (49.4)	173 (47.4)	
Abnormal sperm parameters	584 (50.3)	54 (43.9)	45 (46.4)	202 (51.1)	91 (50.6)	192 (52.6)	
**Infertility type**							0.561
Primary	591 (50.9)	71 (57.7)	46 (47.4)	198 (50.1)	93 (51.7)	183 (50.1)	
Secondary	569 (49.1)	52 (42.3)	51 (52.6)	197 (49.9)	87 (48.3)	182 (49.9)	
**Stimulation protocol**							0.215
GnRH agonist	462 (39.8)	50 (40.7)	47 (48.5)	150 (38.0)	67 (37.2)	148 (40.5)	
GnRH antagonist	686 (59.1)	70 (56.9)	50 (51.5)	242 (61.3)	113 (62.8)	211 (57.8)	
Other	12 (1.0)	3 (2.4)	0 (0.0)	3 (0.8)	0 (0.0)	6 (1.6)	
**Fertilization type**							0.181
IVF	805 (69.4)	92 (74.8)	74 (76.3)	270 (68.4)	128 (71.1)	241 (66.0)	
ICSI	355 (30.6)	31 (25.2)	23 (23.7)	125 (31.6)	52 (28.9)	124 (34.0)	
**Embryo transfer method**							0.407
Fresh	692 (59.7)	79 (64.2)	60 (61.9)	237 (60.0)	93 (51.7)	223 (61.1)	
Frozen	362 (31.2)	36 (29.3)	26 (26.8)	120 (30.4)	69 (38.3)	111 (30.4)	
No transfer	106 (9.1)	8 (6.5)	11 (11.3)	38 (9.6)	18 (10.0)	31 (8.5)	
**Type of embryos transferred**							0.589
Single cleavage-stage embryo	64 (5.5)	7 (5.7)	5 (5.2)	25 (6.3)	10 (5.6)	17 (4.7)	
Double cleavage-stage embryo	759 (65.4)	88 (71.5)	64 (66.0)	255 (64.6)	104 (57.8)	248 (67.9)	
Single blastocyst-stage embryo	213 (18.4)	17 (13.8)	16 (16.5)	72 (18.2)	46 (25.6)	62 (17.0)	
Double blastocyst-stage embryo	18 (1.6)	3 (2.4)	1 (1.0)	5 (1.3)	2 (1.1)	7 (1.9)	
No transfer	106 (9.1)	8 (6.5)	11 (11.3)	38 (9.6)	18 (10.0)	31 (8.5)	

Continuous variables that were not normally distributed were presented as median (25th, 75th percentile) and categorical variables were presented as number (percentage).

MTHFR, 5,10-methylenetetrahydrofolate reductase; PCOS, polycystic ovarian syndrome.

*P* values in bold represent those less than 0.050.

The median (25th, 75th percentile) ages of the included women and their spouses were 33.0 (31.0, 36.0) and 34.0 (31.0, 38.0), respectively; their BMIs were 22.0 (20.2, 24.5) and 25.5 (23.4, 27.8), respectively (Table I). Women with different combined genotypes showed comparable maternal age, paternal age, paternal BMI, maternal cause of infertility, paternal cause of infertility, infertility type, stimulation protocol, fertilization type, embryo transfer method and type of embryos transferred, while they differed significantly in maternal BMI (*P *=* *0.020) (Table I), which would be adjusted as one of the confounders in the later multivariate analyses. Baseline characteristics of women stratified by C677T or A1298C polymorphism were also compared and summarized in Supplementary Tables SIV and SV, respectively.

In addition, we compared the baseline characteristics and overall outcomes between our study population and the other population selected randomly at our center over the same period, who underwent IVF/ICSI treatment but did not receive *MTHFR* genotyping (hereafter referred to as the other IVF/ICSI population). The overall embryological outcomes of our study population were similar to that of the other IVF/ICSI population, while the clinical outcomes were worse in our study population (Supplementary Table SVI), indicating that women at high risk of reproductive failure tended to be referred for *MTHFR* genotyping at our centre.

### Associations between *MTHFR* polymorphisms and the embryological outcomes of IVF/ICSI

**Figure 2.**
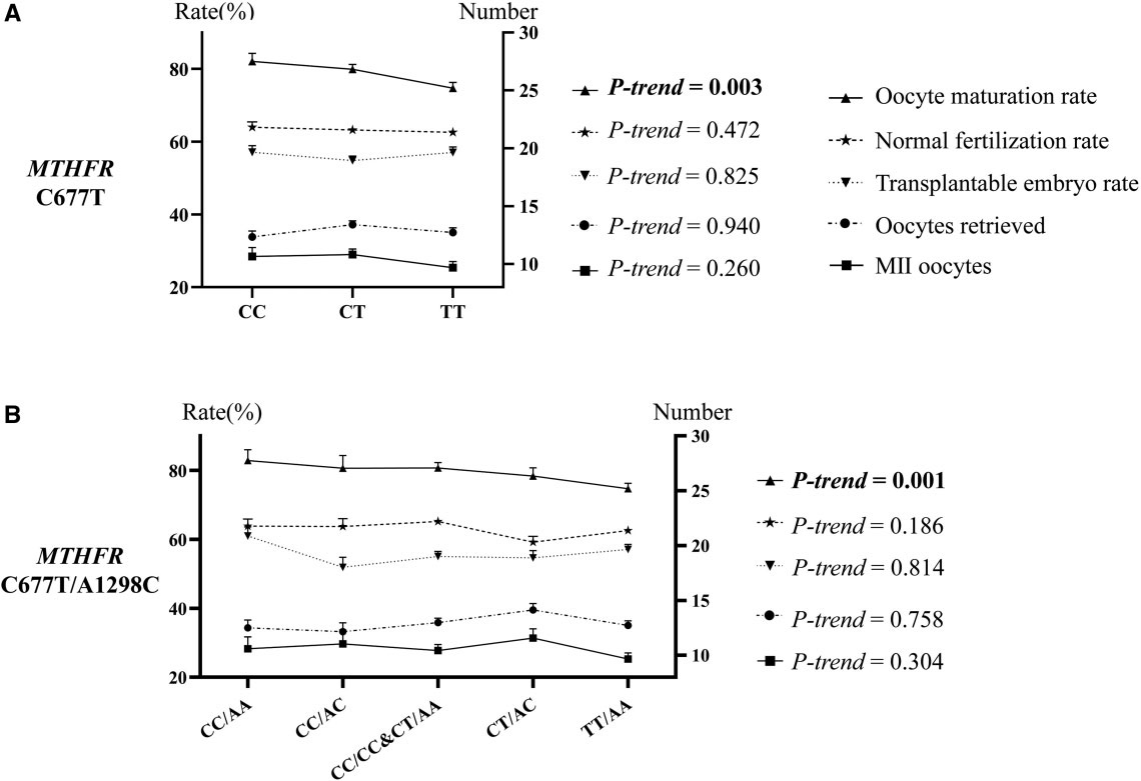
**The trend for each embryological outcome with the activity of the MTHFR enzyme.** The left *Y*-axis indicates the rates of oocyte maturation or normal fertilization or the ratio of the transplantable embryos. The right *Y*-axis indicates the numbers of the oocytes retrieved or the MII oocytes. The *X*-axis indicates the *MTHFR* genotypes. The lines and shapes corresponding to the outcomes are shown in the top right corner. Generalized linear regression models with adjustment for potential confounders were applied to evaluate the linear trend of each outcome with the activity of MTHFR (*P*-trend). *P*-trend values are listed on the right side of each figure. The oocytes retrieved, MII oocytes and oocyte maturation rate were adjusted for maternal age, maternal BMI, maternal cause of infertility, infertility type and stimulation protocol. The normal fertilization rate and transplantable embryo rate were adjusted for maternal age, paternal age, maternal BMI, paternal BMI, maternal cause of infertility, paternal cause of infertility, infertility type, stimulation protocol and fertilization type. (**A**) The trend for each embryological outcome with the MTHFR activity determined by the C677T polymorphism. (**B**) The trend for each embryological outcome with the MTHFR activity determined by the combined C677T/A1298C polymorphism. MII, metaphase II; MTHFR, 5,10-methylenetetrahydrofolate reductase.

To better understand the effect of the combined genotypes, we presented here both the results from analyzing C677T and A1298C separately and in combination. When C677T and A1298C were separately analyzed for their relationship to the embryological outcomes of IVF/ICSI, the oocyte maturation rate was lower in women with the 677TT genotype (n = 365) [74.7% vs. 82.0%, coefficient (95% CI) = −0.07 (−0.13 to −0.02), *P *=* *0.005, Table II and Supplementary Table SVII] and decreased gradually with the reduction of MTHFR activity determined by C677T genotype (*P*-trend = 0.003, Fig. 2A). The C677T polymorphism was not significantly associated with the number of oocytes retrieved or MII oocytes, the normal fertilization rate or the transplantable embryo rate. The A1298C polymorphism was not found to be associated with any embryological outcomes (Table II). Is this the true situation?

**Table II:** Multivariate analysis of the association between *MTHFR* polymorphisms and the embryological outcomes of IVF/ICSI. Oocytes retrieved

*MTHFR* genotypes	**n** ^a^	Oocytes retrieved		Normal fertilization rate		**Transplantable embryo rate**		**n** ^b^	MII oocytes		Oocyte maturation rate	
		Coefficient (95% CI)	*P*-value	Coefficient (95% CI)	*P*-value	Coefficient (95% CI)	*P*-value		Coefficient (95% CI)	*P*-value	Coefficient (95% CI)	*P*-value
**C677T**												
CC	247	Ref.		Ref.		Ref.		64	Ref.		Ref.	
CT	548	0.84 (−0.30 to 1.97)	0.150	−0.01 (−0.04 to 0.03)	0.652	−0.03 (−0.07 to 0.02)	0.257	167	0.23 (−1.43 to 1.89)	0.784	−0.03 (−0.08 to 0.02)	0.280
TT	365	0.19 (−1.04 to 1.41)	0.768	−0.01 (−0.05 to 0.02)	0.469	0.00 (−0.05 to 0.05)	0.985	124	−0.79 (−2.53 to 0.96)	0.378	−0.07 (−0.13 to −0.02)	**0.005**
**A1298C**												
AA	856	Ref.		Ref.		Ref.		270	Ref.		Ref.	
AC&CC	304	0.44 (−0.56 to 1.43)	0.389	−0.03 (−0.06 to 0.00)	0.080	−0.03 (−0.07 to 0.01)	0.133	85	0.60 (−0.83 to 2.02)	0.412	0.00 (−0.04 to 0.05)	0.902
**C677T/A1298C**												
CC/AA	123	Ref.		Ref.		Ref.		31	Ref.		Ref.	
CC/AC	97	−0.40 (−2.42 to 1.62)	0.698	0.00 (−0.06 to 0.06)	0.970	−0.10 (−0.17 to −0.02)	**0.013**	23	0.13 (−3.01 to 3.28)	0.934	−0.05 (−0.14 to 0.05)	0.328
CC/CC&CT/AA	395	0.25 (−1.29 to 1.78)	0.752	0.01 (−0.03 to 0.06)	0.556	−0.06 (−0.12 to −0.01)	**0.030**	125	−0.04 (−2.33 to 2.26)	0.974	−0.04 (−0.10 to 0.03)	0.302
CT/AC	180	1.24 (−0.50 to 2.97)	0.162	−0.05 (−0.10 to 0.01)	0.084	−0.07 (−0.13 to 0.00)	**0.039**	52	0.44 (−2.14 to 3.02)	0.738	−0.07 (−0.14 to 0.01)	0.084
TT/AA	365	−0.04 (−1.58 to 1.51)	0.965	−0.01 (−0.06 to 0.03)	0.613	−0.04 (−0.10 to 0.02)	0.165	124	−0.86 (−3.15 to 1.43)	0.460	−0.09 (−0.16 to −0.03)	**0.007**

Generalized linear regression models with adjustments for potential confounders were applied. Values are coefficient with its 95% CI and *P*-value. Oocytes retrieved, MII oocytes and oocyte maturation rate were adjusted for maternal age, maternal BMI, maternal cause of infertility, infertility type and stimulation protocol. Normal fertilization rate and transplantable embryo rate were adjusted for maternal age, paternal age, maternal BMI, paternal BMI, maternal cause of infertility, paternal cause of infertility, infertility type, stimulation protocol and fertilization type.

a Oocytes retrieved, normal fertilization rate and transplantable embryo rate were analyzed in women treated with IVF and ICSI.

b MII oocytes and oocyte maturation rate were analyzed only in women treated with ICSI.

MII, metaphase II; MTHFR, 5,10-methylenetetrahydrofolate reductase; Ref., reference.

*P* values in bold represent those less than 0.050.

We then analyzed the correlation between the combined C677T/A1298C genotypes and the embryological outcomes. Interestingly, women with the TT/AA genotype (n = 365), whose enzyme activity was the lowest, showed the lowest oocyte maturation rate which was significantly lower than those with the CC/AA genotype (n = 123) [74.7% vs. 82.8%, coefficient (95% CI) = −0.09 (−0.16 to −0.03), *P *=* *0.007, Table II and Supplementary Table SVII]. Remarkably, the oocyte maturation rate showed a linear decline with the reduction of MTHFR activity determined by the combined C677T/A1298C genotype (*P*-trend = 0.001, Fig. 2B). These results suggested that the A1298C polymorphism was not irrelevant to the oocyte maturation rate, but its effect might be biased by the differences in the distribution of C677T genotypes, as described in the Introduction. This speculation was confirmed by the fact that 677TT was distributed only in women with 1298AA genotype, not in women with 1298AC and 1298CC genotypes, which made the proportion of 677TT genotypes significantly higher in women with 1298AA genotype than those with the 1298AC and 1298CC genotypes (42.6% vs. 0%, *P *<* *0.001, Supplementary Table SVIII). Thus, these findings identified a robust relationship between oocyte maturation rate and the combined C677T/A1298C polymorphism.

While the transferable embryo rate did not differ significantly among women with different C677T or A1298C genotypes, differences were found among those with different combined C677T/A1298C genotypes. The transplantable embryo rate was lower in women with CC/AC (n = 97) [51.9%, coefficient (95% CI) = −0.10 (−0.17 to −0.02), *P *=* *0.013], CC/CC&CT/AA (n = 395) [55.0%, coefficient (95% CI) = −0.06 (−0.12 to −0.01), *P *=* *0.030] and CT/AC (n = 180) [54.6%, coefficient (95% CI) = −0.07 (−0.13 to 0.00), *P *=* *0.039] genotypes than those with the CC/AA genotype (n = 123) (61.0%) (Table II and Supplementary Table SVII). Intriguingly, the above four combined genotypes exhibit a moderate, rather than the lowest, MTHFR activity. The number of oocytes retrieved or MII oocytes and the normal fertilization rate were not significantly different between combined variant genotypes and the CC/AA genotype.

### Associations between *MTHFR* polymorphisms and the clinical outcomes of IVF/ICSI

As shown in Table III, although women with the 677TT genotype had an increased likelihood of biochemical pregnancy [aRR = 1.38 (95% CI, 1.04–1.82), *P *=* *0.027, Table III] and live birth [aRR = 2.27 (95% CI, 1.05–4.91), *P *=* *0.037, Table III], the differences were not significant when C677T and A1298C were analyzed in combination. The probability of clinical pregnancy was not significantly different between variant genotypes and the wild-type genotype, whether C677T and A1298C were analyzed separately or jointly (Table III). Moreover, we divided the 221 women achieving clinical pregnancy into two subgroups according to whether they experienced miscarriage, determined the adjusted odds ratio using logistic regression and did not find significant differences between variant genotypes and the wild-type genotype (Supplementary Table SIX). Based on the above results, no significant correlations were observed between combined *MTHFR* C677T/A1298C genotypes and the clinical outcomes of IVF/ICSI in our study population.

**Table III:** Multivariate analysis of the association between *MTHFR* polymorphisms and the clinical outcomes of IVF/ICSI.

*MTHFR* genotypes	Biochemical pregnancy			Clinical pregnancy			Live birth		
	Cases/study subjects (%)	aRR (95% CI)	*P*-value	Cases/study subjects (%)	aRR (95% CI)	*P*-value	Cases/Study subjects (%)	aRR (95% CI)	*P*-value
**C677T**									
CC	53/224 (23.7)	Ref.		42/224 (18.8)	Ref.		8/224 (3.6)	Ref.	
CT	132/496 (26.6)	1.11 (0.84–1.46)	0.465	91/494 (18.4)	0.97 (0.70–1.35)	0.869	28/493 (5.7)	1.61 (0.75–3.46)	0.227
TT	109/334 (32.6)	1.38 (1.04–1.82)	**0.027**	88/334 (26.3)	1.38 (0.99–1.92)	0.052	27/333 (8.1)	2.27 (1.05–4.91)	**0.037**
**A1298C**									
AA	225/783 (28.7)	Ref.		174/782 (22.3)	Ref.		51/781 (6.5)	Ref.	
AC&CC	69/271 (25.5)	0.89 (0.71–1.12)	0.313	47/270 (17.4)	0.80 (0.60–1.07)	0.126	12/269 (4.5)	0.71 (0.39–1.31)	0.272
**C677T/A1298C**									
CC/AA	26/115 (22.6)	Ref.		22/115 (19.1)	Ref.		4/115 (3.5)	Ref.	
CC/AC	23/86 (26.7)	1.17 (0.72–1.91)	0.516	17/86 (19.8)	1.02 (0.58–1.80)	0.939	4/86 (4.7)	1.26 (0.33–4.84)	0.742
C/CC&CT/AA	94/357 (26.3)	1.15 (0.79–1.68)	0.471	67/356 (18.8)	0.97 (0.63–1.50)	0.889	20/356 (5.6)	1.52 (0.53–4.33)	0.437
CT/AC	42/162 (25.9)	1.13 (0.74–1.73)	0.564	27/161 (16.8)	0.88 (0.53–1.47)	0.626	8/160 (5.0)	1.51 (0.47–4.88)	0.495
TT/AA	109/334 (32.6)	1.44 (0.99–2.08)	0.055	88/334 (26.3)	1.36 (0.89–2.05)	0.152	27/333 (8.1)	2.25 (0.81–6.25)	0.122

The clinical outcomes were compared using log-binomial regression models and the RRs were adjusted for maternal age, maternal BMI, maternal cause of infertility, paternal cause of infertility, infertility type, fertilization type, embryo transfer method and type of embryos transferred.

aRR, adjusted risk ratio; MTHFR, 5,10-methylenetetrahydrofolate reductase; Ref., reference.

*P* values in bold represent those less than 0.050.

## Discussion

The present study systematically and carefully explored the associations of the combined *MTHFR* C677T/A1298C genotypes with the IVF/ICSI outcomes and revealed some interesting findings. Our results for the first time showed that the oocyte maturation rate decreased linearly with the reduction of MTHFR activity determined by combined C677T/A1298C polymorphisms, while the genotypes with the intermediate enzyme activity were related to a worse Day 3 embryo quality.

Although D'Elia et al. (2014) found that patients with the 677CT+TT genotype had a lower proportion of mature oocytes than those with the wild-type genotype, this difference was no longer significant when patients were roughly divided into three groups stratified by C677T and A1298C (no polymorphism, one polymorphism and both polymorphisms), which might be due to its limited sample size (82 patients). In our current study, as many as 1160 women were investigated. In addition, we studied the relationship between each combined C677T/A1298C genotype and the oocyte maturation rate, thus revealing for the first time that the oocyte maturation rate decreased with the reduction of MTHFR activity.

The development of human oocytes is actually a long and complex process of folliculogenesis. The primordial follicle pool is established before birth, and after a dozen years of dormancy, primordial follicles are activated from puberty and then enter the growth period. It takes about 300 days for primordial follicles to form preantral follicles. After 70 additional days, some follicles reach a size of 2–5 mm, which are referred to as selectable follicles. In women of reproductive age, there are ∼3–11 selectable follicles per ovary. Physiologically, only one follicle with the most rapid growth and the most sensitive to FSH per menstrual cycle can be selected as the dominant follicle to continue developing, while the other follicles go through atresia. The dominant follicle dramatically grows to 18–20 mm in the next 15–20 days, becoming a preovulatory follicle (Gougeon, 1986, 2010). The main changes during the growth period are: (i) oocyte growth and maturation and expression of key genes, (ii) granulosa cell proliferation (from a few to tens of millions), forming gap junctions with oocytes and expression of FSH receptors, (iii) theca cell proliferation and expression of LH receptors, (iv) estrogen synthesis and secretion by granulosa cells and theca cells and (v) follicular fluid production and accumulation (Gougeon, 1986, 2010). Previous studies have confirmed that: (i) folate and homocysteine are present in follicular fluid and their concentrations are positively correlated with that of their corresponding substance in serum (Steegers-Theunissen *et al.*, 1993), (ii) folic acid supplementation can increase folate and decrease homocysteine levels in follicular fluid (Boxmeer *et al.*, 2008) and (iii) in follicular fluid, the homocysteine level is negatively correlated with the folate level, fertilization rate and embryo quality in PCOS patients (Berker *et al.*, 2009). Based on the above evidence, the decrease in MTHFR enzyme activity mediated by C677T and A1298C polymorphisms may impair folliculogenesis by: (i) suppressing DNA and protein synthesis and then inhibiting the proliferation of granulosa cells and theca cells, (ii) altering the DNA methylation pattern of oocytes and disrupting the expression of crucial genes for folliculogenesis and/or (iii) creating an adverse microenvironment by elevating homocysteine concentrations and decreasing folate concentrations in follicular fluid. Variants of *MTHFR* may cause detrimental effects on oocytes at different stages of folliculogenesis in one or more ways, especially during the growth period. Therefore, women with *MTHFR* variants may have a higher proportion of poorly developed follicles. During natural pregnancy, only one dominant follicle grows per cycle, follicles suffering more from metabolic defects caused by *MTHFR* variants are less likely to be the dominant follicle, so the adverse effects of *MTHFR* variants may not be apparent. However, ovarian stimulation during IVF/ICSI treatment activates follicles that should have been undergoing atresia, some of which may be the poor-quality follicles affected by the *MTHFR* variants. Moreover, the huge increase in metabolic demands following the simultaneous activation of multiple follicles may further exacerbate the metabolic defects in these follicles, ultimately resulting in a lower oocyte maturation rate. The exact mechanism of the effect of *MTHFR* polymorphisms on oocyte maturation during IVF/ICSI treatment needs to be further explored.

Oocyte maturation is important for subsequent embryonic development. Human zygotic genome activation occurs at the 4–8 cell stage (Jukam *et al.*, 2017) and embryonic development is regulated by maternal genes of oocytes from zygotes to Day 3 embryos. The adverse effects of *MTHFR* polymorphisms may result in some oocytes with low MTHFR enzyme activity possessing poor developmental potential. The lower the activity, the more likely they will show abnormality and be eliminated at earlier stages. The remaining oocytes/embryos are more likely to show near-normal development potential. This may be the reason why the TT/AA genotype with the lowest enzyme activity had a lower oocyte maturation rate but an analogous Day 3 embryo quality, while the CC/AC, CT/AA&CC/CC and CT/AC genotypes with intermediate enzyme activity presented a comparable oocyte maturation rate but a lower Day 3 embryo quality. The elucidation of this intricate relationship also benefited from the large sample size in our study and the assignment of cohorts according to MTHFR activity determined by the combined C677T/A1298C genotypes, unlike previous studies that did not find a relationship between *MTHFR* polymorphisms and embryo quality (Dobson *et al.*, 2007; Marci *et al.*, 2012; D'Elia *et al.*, 2014).

Despite the above-mentioned strengths, our study did not find an association between combined C677T/A1298C genotypes and the clinical outcomes of IVF/ICSI, and the reasons for this are intricate. First, during IVF/ICSI treatment, embryos of better quality are artificially selected for transfer, thereby reducing the differences in developmental potential between embryos with different maternal *MTHFR* genotypes. Second, all of our study participants took folic acid 3 months before pregnancy, so there was sufficient folic acid for post-implantation embryo development, which further weakened the effect of *MTHFR* polymorphisms. Third, fetal intrauterine development is affected by many other factors such as placental function (Maltepe and Fisher, 2015; Aplin *et al.*, 2020), which increases the complexity of studying the clinical outcomes of fetal development. Probably for the same reasons, other studies have also failed to reveal the relationship between maternal *MTHFR* polymorphisms and clinical outcomes of IVF/ICSI (Dobson *et al.*, 2007; Marci *et al.*, 2012; D'Elia *et al.*, 2014; Murto *et al.*, 2015; Patounakis *et al.*, 2016). In addition, paternal *MTHFR* polymorphisms may have an impact on clinical outcomes, interfering with the effects of maternal *MTHFR* polymorphisms. Our results would not be affected as long as there is no significant difference in the distribution of paternal *MTHFR* polymorphisms between the cohorts we studied. Unfortunately, as males are not routinely recommended for *MTHFR* genotyping, we do not have sufficient data to assess the impact of paternal *MTHFR* polymorphisms on clinical outcomes. A study conducted in 197 IVF cycles showed that maternal *MTHFR* C677T and A1298C genotypes did not affect any of the clinical outcomes (pregnancy rate, spontaneous abortion rate, etc.) when controlling for the male genotypes (Dobson *et al.*, 2007), and their results are consistent with ours. A larger cohort study, more strictly controlling for eligibility criteria and confounders in more participants, may help uncover the potential impact of *MTHFR* polymorphisms on the clinical outcomes of IVF/ICSI.

Although we did not find an association of *MTHFR* polymorphisms with the clinical outcomes of IVF/ICSI, our findings regarding the association of *MTHFR* polymorphisms with oocyte maturation and embryo quality have important implications. Numerous studies, including ours, have demonstrated that oocytes and early embryos undergo dramatic and extensive DNA methylation reprogramming (Guo *et al.*, 2014; Smith *et al.*, 2014; Sendžikaitė and Kelsey, 2019; Zhou *et al.*, 2019; Yan *et al.*, 2021), which may be perturbed by polymorphisms of *MTHFR*, a key enzyme in methyl-donor metabolism, thereby leading to potential epigenetic abnormalities in oocytes and embryos in early life. According to the theory of ‘Gamete and Embryo-fetal Origins of Adult Diseases (GEOAD)’, the disturbance of DNA methylation pattern of oocytes or embryos is not only related to the health of offspring after birth, such as a high risk of hypertension, diabetes, cancer or neurological impairment, but also has a profound effect on the health of grandoffspring via transgenerational epigenetic effects (Zou *et al.*, 2019). Padmanabhan *et al.* (2013) demonstrated that mutation of the mouse folate metabolism gene *Mtrr* in maternal grandparents was sufficient to cause defects including intrauterine growth restriction and congenital malformations in their wild-type grandoffspring. Therefore, whether maternal *MTHFR* polymorphism has an impact on the postnatal health of offspring or the health of the grandoffspring is worth further exploration.

A limitation of our study is that we did not obtain the folate concentration of each patient. However, we analyzed the available serum folate (103 cases) and homocysteine (217 cases) concentrations during the peri-conception period in our participants (Supplementary Tables SX and SXI). The results showed that the folate levels of women with *MTHFR* variants were lower than those of the wild-type genotype, while the homocysteine concentrations are quite close among women with different genotypes, suggesting that folic acid supplementation could compensate for the metabolic defects to some extent. This result implied that adequate folate may attenuate the effect of *MTHFR* variants on IVF/ICSI clinical outcomes. Moreover, this result was consistent with our above view about the impact of *MTHFR* variants on embryological outcomes. In the early stages of folliculogenesis (1 year before ovulation and earlier), when most people do not take folic acid supplements, *MTHFR* variants are associated with lower basal folate levels (Tsang *et al.*, 2015) and may lead to compromised follicle development in multiple ways described above. During the peri-conception period, our participants were all advised to take folic acid, so both women with and without *MTHFR* variants had elevated folate levels, but the latter women still had higher levels than the former women. For natural pregnancy, this difference might be imperceptible in the only dominant follicle. However, it would be pronounced for women undergoing ovarian stimulation, as we discussed earlier. The situations at different stages of folliculogenesis together resulted in a poor oocyte quality in women with *MTHFR* variants. Therefore, the available results of folate and homocysteine concentrations further supported our above statement regarding the association of *MTHFR* variants with the embryological and clinical outcomes of IVF/ICSI.

**Figure 3.**
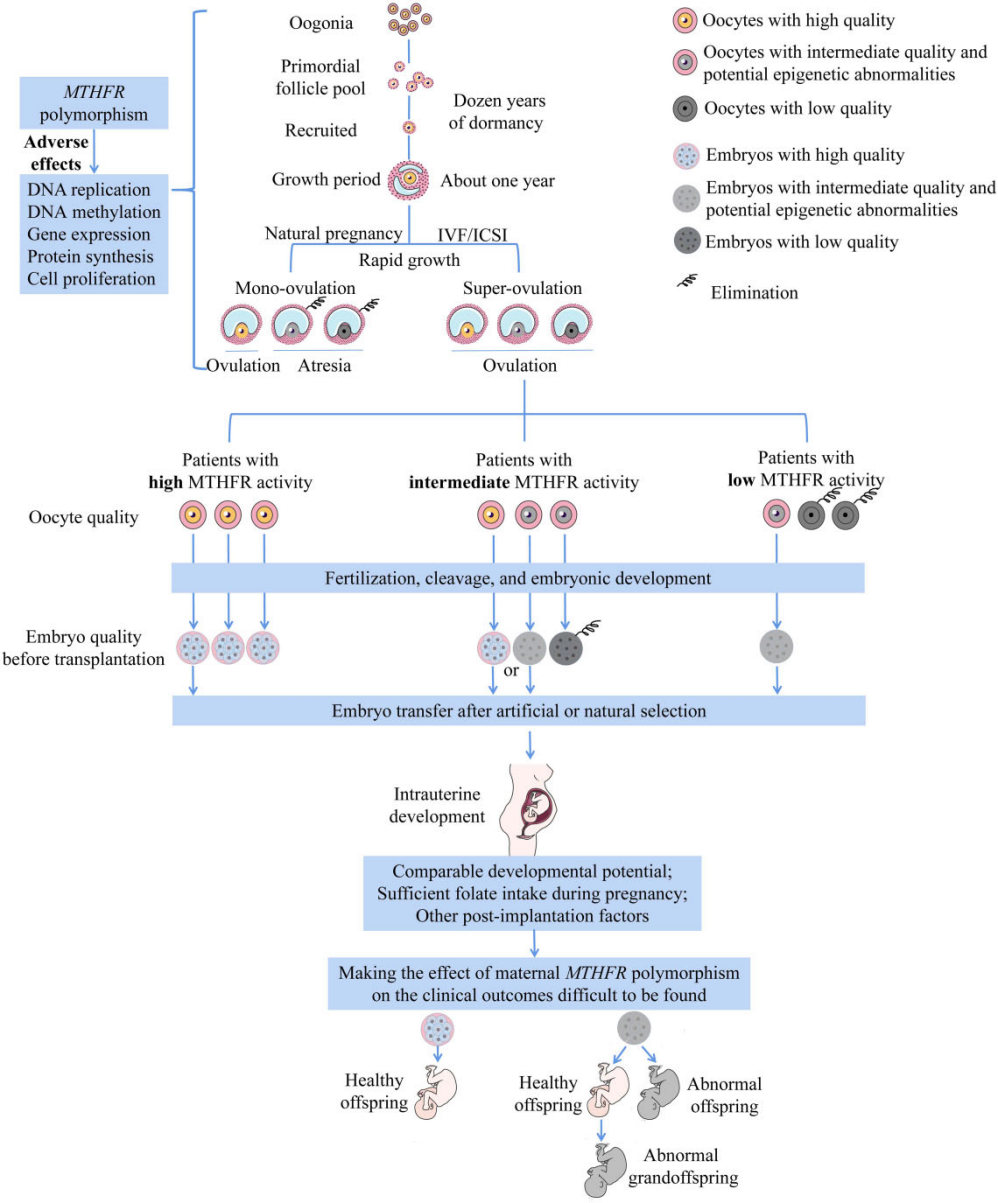
**Schematic overview of putative ways in which *MTHFR* polymorphisms affect reproductive health**. MTHFR, 5,10-methylenetetrahydrofolate reductase.

In conclusion, our study provided a comprehensive insight into the intricate relationship between the *MTHFR* C677T and A1298C polymorphisms and the embryological and clinical outcomes of IVF/ICSI in women referred for *MTHFR* genotyping. Based on our results and available knowledge, we proposed putative ways how *MTHFR* polymorphisms affected the IVF/ICSI outcomes, as summarized in Fig. 3. *MTHFR* polymorphisms have weak but persistent adverse effects on DNA replication and methylation, gene expression, protein synthesis and cell proliferation in the process of folliculogenesis. Ovarian stimulation during IVF/ICSI treatment dramatically increases the metabolic demands of oocytes, making the metabolic defects caused by *MTHFR* variants more pronounced. Therefore, oocytes with low MTHFR enzyme activity have poor oocyte quality (i.e. low maturation rate and potential epigenetic abnormalities). Due to the reasons discussed above, women with the lowest enzyme activity have the poorest oocyte maturation potential, while those with intermediate enzyme activity presented a lower Day 3 embryo quality. Ultimately, the normal embryos or those with potential epigenetic abnormalities are implanted. These embryos may possess a comparable potential for intrauterine development, which, together with sufficient folate intake during pregnancy and other post-implantation factors such as placental function, makes the effect of maternal *MTHFR* polymorphisms on the clinical outcomes difficult to find. However, maternal *MTHFR* polymorphisms may have profound effects on offspring and even grandoffspring by altering epigenetic patterns. Thus, our study not only elucidates the relationship between *MTHFR* polymorphisms and the oocyte/embryo quality but also provides novel directions and clues for future research.

## Supplementary Material

hoac055_Supplementary_Data

## Data Availability

The data underlying this article will be shared on reasonable request to the corresponding author.
