# Supplementary material for: Association between maternal MTHFR C677T/A1298C combination polymorphisms and IVF/ICSI outcomes: a retrospective cohort study
Source: Hum Reprod Open. 2022 Dec 5;2023(1):hoac055. doi: 10.1093/hropen/hoac055 (PMC9749479; doi:10.1093/hropen/hoac055)
Supplement: hoac055_Supplementary_Data [file hoac055_supplementary_data.docx]

**Supplementary Table SI. The homocysteine concentration (μmol/L) of 3923 women with different combined *MTHFR* 677/1298 genotypes**

| ***MTHFR* genotype** | **adjusted**  **(Mean±SEM)** | **Coefficient** | ***P*-trend** |
| --- | --- | --- | --- |
| C677T/A1298C |  |  |  |
| CC/AA(n=350) | 6.67±0.23 | 0.694 (0.587~0.800) | <0.001 |
| CC/AC(n=327) | 6.69±0.24 |  |  |
| CC/CC&CT/AA(n=1325) | 7.16±0.12 |  |  |
| CT/AC(n=634) | 7.19±0.17 |  |  |
| TT/AA(n=1287) | 9.13±0.12 |  |  |

Generalized linear regression models were used to calculate the adjusted (Mean±SEM), coefficient, and *P*-trend which were adjusted for age and diagnosis.

MTHFR=5,10-methylenetetrahydrofolate reductase; SEM: standard error of the means.

**Supplementary Table SII. Comparison of *MTHFR* genotype distribution between our study population and the general Han women in northern China (Data were summarized from Wang et al.**)

| ***MTHFR* genotype** | | **Our study population n(%)** | **Han women in Northern China n(%)** | **χ^2^** | ***P* value** |
| --- | --- | --- | --- | --- | --- |
| C677T | |  |  |  |  |
|  | CC | 247(21.3) | 4019(20.3) | 5.071 | 0.079 |
|  | CT | 548(47.2) | 8887(45.0) |  |  |
|  | TT | 365(31.5) | 6858(34.7) |  |  |
|  | Allele C | 1042(44.9) | 16925(42.8) | 3.93 | 0.050 |
|  | Allele T | 1278(55.1) | 22603(57.2) |  |  |
| A1298C | |  |  |  |  |
|  | AA | 856(73.8) | 14065(71.7) | 3.593 | 0.165 |
|  | AC | 277(23.9) | 5155(26.3) |  |  |
|  | CC | 27(2.3) | 396(2.0) |  |  |
|  | Allele A | 1989(85.7) | 33285(84.8) | 1.357 | 0.245 |
|  | Allele C | 331(14.3) | 5947(15.2) |  |  |

MTHFR=5,10-methylenetetrahydrofolate reductase.

**Supplementary Table SIII. Hardy-Weinberg equilibrium test of *MTHFR* genotypes**

| ***MTHFR* genotype** | | **Actual frequency n(%)** | **Expected frequency n(%)** | **χ^2^** | ***P* value** |
| --- | --- | --- | --- | --- | --- |
| C677T | |  |  |  |  |
|  | CC | 247(21.3) | 234(20.2) |  |  |
|  | CT | 548(47.2) | 574(49.5) |  |  |
|  | TT | 365(31.5) | 352(30.3) | 1.190 | 0.550 |
|  | Allele C | 1042(44.9) |  |  |  |
|  | Allele T | 1278(55.1) |  |  |  |
| A1298C | |  |  |  |  |
|  | AA | 856(73.8) | 853(73.5) |  |  |
|  | AC | 277(23.9) | 284(24.5) |  |  |
|  | CC | 27(2.3) | 23(2.0) | 0.269 | 0.870 |
|  | Allele A | 1989(85.7) |  |  |  |
|  | Allele C | 331(14.3) |  |  |  |

MTHFR=5,10-methylenetetrahydrofolate reductase.

**Supplementary Table SIV. Baseline characteristics of participants with different *MTHFR* C677T genotypes**

| **Characteristic** | CC | CT | TT | P value |
| --- | --- | --- | --- | --- |
| **n(%)** | 247(21.3) | 548(47.2) | 365(31.5) |  |
| **Maternal age, years** | 34.0 (31.0, 36.5) | 33.0 (31.0, 36.0) | 33.0 (30.0, 36.0) | 0.031 |
| **Paternal age, years** | 34.0 (32.0, 38.0) | 34.0 (31.0, 37.0) | 34.0 (30.0, 38.0) | 0.355 |
| **Maternal BMI, kg/m2** | 21.8 (20.2, 23.9) | 21.9 (20.2, 24.2) | 22.1 (20.3, 25.4) | 0.080 |
| **Paternal BMI, kg/m2** | 25.1 (23.5, 27.1) | 25.6 (23.2, 28.1) | 25.5 (23.4, 27.5) | 0.235 |
| **Maternal cause of infertility** |  |  |  | 0.649 |
| Unexplained | 78（31.6） | 163（29.7） | 108（29.6） |  |
| Tubal factor | 83（33.6） | 174（31.8） | 113（31.0） |  |
| PCOS | 21（8.5） | 69（12.6） | 48（13.2） |  |
| Diminished ovarian reserve | 34（13.8） | 70（12.8） | 45（12.3） |  |
| Endometriosis | 15（6.1） | 25（4.6） | 14（3.8） |  |
| Other | 16（6.5） | 47（8.6） | 37（10.1） |  |
| **Paternal cause of infertility** |  |  |  | 0.285 |
| Unexplained | 133（53.8） | 270（49.3） | 173（47.4） |  |
| Abnormal sperm parameters | 114（46.2） | 278（50.7） | 192（52.6） |  |
| **Infertility type** |  |  |  | 0.824 |
| Primary | 130（52.6） | 278（50.7） | 183（50.1） |  |
| Secondary | 117（47.4） | 270（49.3） | 182（49.9） |  |
| **Stimulation protocol** |  |  |  | 0.233 |
| GnRH agonist | 107（43.3） | 207（37.8） | 148（40.5） |  |
| GnRH antagonist | 137（55.5） | 338（61.7） | 211（57.8） |  |
| Other | 3（1.2） | 3（0.5） | 6（1.6） |  |
| **Fertilization type** |  |  |  | 0.105 |
| IVF | 183（74.1） | 381（69.5） | 241（66.0） |  |
| ICSI | 64（25.9） | 167（30.5） | 124（34.0） |  |
| **Embryo transfer method** |  |  |  | 0.573 |
| Fresh | 155（62.8） | 314（57.3） | 223（61.1） |  |
| Frozen | 69（27.9） | 182（33.2） | 111（30.4） |  |
| No transfer | 23（9.3） | 52（9.5） | 31（8.5） |  |
| **Type of embryos transferred** |  |  |  | 0.474 |
| Single cleavage-stage embryo | 14（5.7） | 33（6.0） | 17（4.7） |  |
| Double cleavage-stage embryo | 170（68.8） | 341（62.2） | 248（67.9） |  |
| Single blastocyst-stage embryo | 36（14.6） | 115（21.0） | 62（17.0） |  |
| Double blastocyst-stage embryo | 4（1.6） | 7（1.3） | 7（1.9） |  |
| No transfer | 23（9.3） | 52（9.5） | 31（8.5） |  |

Continuous variables that were not normally distributed were presented as median (25th, 75th percentile) and categorical variables were reported as number (percentage).

MTHFR=5,10-methylenetetrahydrofolate reductase; BMI=body mass index; PCOS=polycystic ovarian syndrome; IVF=*in vitro* fertilization; ICSI=intracytoplasmic sperm injection .

**Supplementary Table SV. Baseline characteristics of participants with different *MTHFR* A1298C genotypes**

| **Characteristic** | AA | AC&CC | *P* value |
| --- | --- | --- | --- |
| **n(%)** | 856(73.8) | 304(26.2) |  |
| **Maternal age, years** | 33.0 (30.0, 36.0) | 33.0 (31.0, 36.0) | 0.063 |
| **Paternal age, years** | 34.0 (31.0, 37.0) | 34.0 (32.0, 38.0) | 0.052 |
| **Maternal BMI, kg/m2** | 22.0 (20.1, 24.5) | 22.0 (20.3, 24.6) | 0.582 |
| **Paternal BMI, kg/m2** | 25.5 (23.4, 27.7) | 25.6 (23.4, 27.8) | 0.356 |
| **Maternal cause of infertility** |  |  | 0.578 |
| Unexplained | 250（29.2） | 99（32.6） |  |
| Tubal factor | 267（31.2） | 103（33.9） |  |
| PCOS | 106（12.4） | 32（10.5） |  |
| Diminished ovarian reserve | 115（13.4） | 34（11.2） |  |
| Endometriosis | 40（4.7） | 14（4.6） |  |
| Other | 78（9.1） | 22（7.2） |  |
| **Paternal cause of infertility** |  |  | 0.784 |
| Unexplained | 423（49.4） | 153（50.3） |  |
| Abnormal sperm parameters | 433（50.6） | 151（49.7） |  |
| **Infertility type** |  |  | 0.700 |
| Primary | 439（51.3） | 152（50.0） |  |
| Secondary | 417（48.7） | 152（50.0） |  |
| **Stimulation protocol** |  |  | 0.113 |
| GnRH agonist | 338（39.5） | 124（40.8） |  |
| GnRH antagonist | 506（59.1） | 180（59.2） |  |
| Other | 12（1.4） | 0（0.0） |  |
| **Fertilization type** |  |  | 0.244 |
| IVF | 586（68.5） | 219（72.0） |  |
| ICSI | 270（31.5） | 85（28.0） |  |
| **Embryo transfer method** |  |  | 0.202 |
| Fresh | 523（61.1） | 169（55.6） |  |
| Frozen | 260（30.4） | 102（33.6） |  |
| No transfer | 73（8.5） | 33（10.9） |  |
| **Type of embryos transferred** |  |  | 0.252 |
| Single cleavage-stage embryo | 47（5.5） | 17（5.6） |  |
| Double cleavage-stage embryo | 573（66.9） | 186（61.2） |  |
| Single blastocyst-stage embryo | 148（17.3） | 65（21.4） |  |
| Double blastocyst-stage embryo | 15（1.8） | 3（1.0） |  |
| No transfer | 73（8.5） | 33（10.9） |  |

Continuous variables that were not normally distributed were presented as median (25th, 75th percentiles) and categorical variables were reported as number (percentage).

MTHFR=5,10-methylenetetrahydrofolate reductase; BMI=body mass index; PCOS=polycystic ovarian syndrome; IVF=in vitro fertilization; ICSI=intracytoplasmic sperm injection .

**Supplementary Table SVI. Comparison between our study population and the other IVF/ICSI population at our center over the same period**

| **Characteristic** | **Our study population （n=1160) Mean±SD or n(%)** | **The other IVF/ICSI population  (n=2469) Mean±SD or n(%)** | ***P* value** |
| --- | --- | --- | --- |
| **Maternal age, years** | 33.0(31.0, 36.0) | 32.0(29.0, 35.0) | <0.001 |
| **Paternal age, years** | 34.0(31.0, 38.0) | 33.0(30.0, 37.0) | <0.001 |
| **Maternal BMI, kg/m2** | 22.0(20.2, 24.5) | 22.1(20.2, 24.7) | 0.159 |
| **Paternal BMI, kg/m2** | 25.5(23.4, 27.8) | 25.2(22.9, 27.7) | 0.042 |
| **Maternal cause of infertility** |  |  |  |
| None | 349(30.1) | 658(26.7) |  |
| Tubal factor | 370(31.9) | 841(34.1) |  |
| PCOS | 138(11.9) | 308(12.5) |  |
| Diminished ovarian reserve | 149(12.8) | 326(13.2) |  |
| Endometriosis | 54(4.7) | 139(5.6) |  |
| Other | 100(8.6) | 197(8.0) | 0.255 |
| **Paternal cause of infertility** |  |  |  |
| None | 576(49.7) | 1028(41.6) |  |
| Abnormal sperm parameters | 584(50.3) | 1441(58.4) | <0.001 |
| **Infertility type** |  |  |  |
| Primary | 591(50.9) | 1467(59.4) |  |
| Secondary | 569(49.1) | 1002(40.6) | <0.001 |
| **Stimulation Protocol** |  |  |  |
| GnRH agonist | 462(39.8) | 887(35.9) |  |
| GnRH antagonist | 686(59.1) | 1518(61.5) |  |
| Other | 12(1.0) | 64(2.6) | 0.001 |
| **Fertilization type** |  |  |  |
| IVF | 805(69.4) | 1666(67.5) |  |
| ICSI | 355(30.6) | 803(32.5) | 0.252 |
| **Embryo transfer method** |  |  |  |
| Fresh | 692(59.7) | 1480(59.9) |  |
| Frozen | 362(31.2) | 733(29.7) |  |
| No transfer | 106(9.1) | 256(10.4) | 0.405 |
| **Type of embryos transferred** |  |  |  |
| Single cleavage-stage embryo | 64(5.5) | 214(8.7) |  |
| Double cleavage-stage embryo | 759(65.4) | 1608(65.1) |  |
| Single blastocyst-stage embryo | 213(18.4) | 383(15.5) |  |
| Double blastocyst-stage embryo | 18(1.6) | 8(0.3) |  |
| No transfer | 106(9.1) | 256(10.4) | <0.001 |
| **Embryological outcomes** |  |  |  |
| Oocytes retrieved | 13.0±8.2 | 12.3±7.7 | 0.032 |
| MⅡ oocytes | 10.4±6.4 | 10.1±6.0 | 0.714 |
| Oocyte maturation rate(%) | 78.4±18.0 | 78.3±18.7 | 0.886 |
| Normal fertilization rate(%) | 63.1±22.7 | 63.3±24.0 | 0.647 |
| Transplantable embryo rate(%) | 56.0±28.7 | 57.8±29.1 | 0.062 |
| **Clinical outcomes** |  |  |  |
| Biochemical pregnancy | 294/1054(27.9) | 1085/2213(49.0) | <0.001 |
| Clinical pregnancy | 221/1052(21.0) | 975/2209(44.1) | <0.001 |
| Miscarriage | 150/199(75.4) | 138/975(14.2) | <0.001 |
| Live birth | 63/1050(6.0) | 652/2047(31.9) | <0.001 |
| Preterm birth | 10/64 (15.6) | 92/653(14.1) | 0.737 |

IVF=in-vitro fertilization; ICSI=intracytoplasmic sperm injection; SD=standard deviation; BMI=body mass index; PCOS=polycystic ovarian syndrome.

**Supplementary Table SVII. The mean and adjusted mean of each embryological outcome of IVF/ICSI**

| **MTHFR genotype** | **n^a^** | **Oocytes retrieved** | | **Normal fertilization rate(%)** | | **Transplantable embryo rate(%)** | | **n^b^** | **MⅡ oocytes** | | **Oocyte maturation rate(%)** | |
| --- | --- | --- | --- | --- | --- | --- | --- | --- | --- | --- | --- | --- |
|  |  | Mean±SEM | adjusted (Mean±SEM) | Mean±SEM | adjusted (Mean±SEM) | Mean±SEM | adjusted (Mean±SEM) |  | Mean±SEM | adjusted (Mean±SEM) | Mean±SEM | adjusted (Mean±SEM) |
| **C677T** | | | | | | | | | | | | |
| CC | 247 | 12.1±0.2 | 12.3±0.5 | 63.8±1.3 | 64.0±1.4 | 56.7±1.8 | 57.1±1.8 | 64 | 10.8±0.9 | 10.7±0.8 | 81.8±1.8 | 82.0±2.2 |
| CT | 548 | 13.5±0.4 | 13.4±0.3 | 63.2±1.0 | 63.2±1.0 | 54.8±1.2 | 54.8±1.2 | 167 | 10.9±0.5 | 10.8±0.5 | 80.1±1.4 | 79.9±1.4 |
| TT | 365 | 12.8±0.4 | 12.7±0.4 | 62.6±1.2 | 62.6±1.2 | 57.3±1.5 | 57.0±1.5 | 124 | 9.5±0.5 | 9.7±0.5 | 74.5±1.7 | 74.7±1.6 |
| **A1298C** | | | | | | | | | | | | |
| AA | 856 | 12.9±0.3 | 12.8±0.3 | 63.9±0.8 | 63.8±0.8 | 56.7±1.0 | 56.7±1.0 | 270 | 10.2±0.4 | 10.1±0.4 | 78.2±1.1 | 78.1±1.1 |
| AA+AC | 304 | 13.2±0.5 | 13.3±0.5 | 61.0±1.3 | 61.2±1.3 | 53.8±1.6 | 54.0±1.6 | 85 | 11.1±0.8 | 11.2±0.7 | 79.4±1.7 | 79.4±1.9 |
| **C677T/A1298C** | | | | | | | | | | | | |
| CC/AA | 123 | 12.3±0.7 | 12.5±0.7 | 63.8±1.7 | 63.8±2.0 | 60.5±2.5 | 61.0±2.6 | 31 | 11.1±1.2 | 10.6±1.1 | 82.5±2.7 | 82.8±3.2 |
| CC/AC | 97 | 11.9±0.8 | 12.2±0.8 | 63.2±2.4 | 63.7±2.3 | 51.6±2.9 | 51.9±2.9 | 23 | 11.3±1.8 | 11.0±1.3 | 80.3±2.7 | 80.6±3.7 |
| CC/CC&CT/AA | 395 | 13.0±0.4 | 13.0±0.4 | 65.3±1.1 | 65.2±1.1 | 55.2±1.5 | 55.0±1.4 | 125 | 10.4±0.6 | 10.4±0.5 | 81.0±1.6 | 80.7±1.6 |
| CT/AC | 180 | 14.2±0.7 | 14.1±0.6 | 59.0±1.8 | 59.2±1.7 | 54.4±2.1 | 54.6±2.1 | 52 | 11.5±0.9 | 11.6±0.8 | 78.3±2.4 | 78.3±2.4 |
| TT/AA | 365 | 12.8±0.4 | 12.7±0.4 | 62.6±1.2 | 62.5±1.2 | 57.3±1.5 | 57.1±1.5 | 124 | 9.5±0.5 | 9.7±0.5 | 74.5±1.7 | 74.7±1.6 |

Adjusted (Mean±SEM) was calculated by performing a generalized linear regression model with adjustment for potential confounders. Oocytes retrieved, MⅡoocytes, and oocyte maturation rate were adjusted for maternal age, maternal BMI, maternal cause of infertility, infertility type, and stimulation protocol. Normal fertilization rate and transplantable embryo rate were adjusted for maternal age, paternal age, maternal BMI, paternal BMI, maternal cause of infertility, paternal cause of infertility, infertility type, stimulation protocol, and fertilization type.

^a^Oocytes retrieved, normal fertilization rate, and transplantable embryo rate were analyzed in women treated with IVF and ICSI.

^b^MⅡ oocytes and oocyte maturation rate were analyzed only in women treated with ICSI.

MTHFR=5,10-methylenetetrahydrofolate reductase; IVF=in-vitro fertilization; ICSI=intracytoplasmic sperm injection. SEM: standard error of the mean.

**Supplementary Table SVIII. Different distribution of 677TT genotype in women with 1298AA and 1298AC&CC genotype**

|  |  | **A1298C** | | **P value** |
| --- | --- | --- | --- | --- |
|  |  | **AA** | **AC&CC** |  |
| **C677T** | **CC&CT** | 491(57.4) | 304(100.0) | <0.001 |
|  | **TT** | 365(42.6) | 0(0.0) |  |

**Supplementary Table SIX. Multivariate logistic regression analysis between two subgroups of our study population stratified by miscarriage/no miscarriage**

| **Characteristic** | **Miscarriage**  **(n=150)** | **No miscarriage**  **(n=69)** | **aOR(95%CI)** | ***P* value** |
| --- | --- | --- | --- | --- |
|  | Mean±SD or n(%) | Mean±SD or n(%) |  |  |
|  |  |  |  |  |
| **Maternal age, years** | 33.1±3.8 | 32.6±3.3 | 1.06(0.97~1.16) | 0.174 |
| **Maternal BMI,kg/m2** | 22.8±3.5 | 21.7±2.7 | 1.11(1.00~1.23) | 0.044 |
| **Maternal cause of infertility** |  |  |  |  |
| None | 42(28.0) | 23(33.3) | 1 |  |
| Tubal factor | 46(30.7) | 26(37.7) | 1.04(0.48~2.27) | 0.925 |
| PCOS | 24(16.0) | 6(8.7) | 2.13(0.70~6.50) | 0.183 |
| Diminished ovarian reserve | 18(12.0) | 8(11.6) | 0.98(0.33~2.87) | 0.967 |
| Endometriosis | 8(5.3) | 3(4.3) | 1.55(0.35~6.83) | 0.564 |
| Other | 12(8.0) | 3(4.3) | 2.03(0.48~8.55) | 0.335 |
| **Paternal cause of infertility** |  |  |  |  |
| None | 70(46.7) | 29(42.0) | 1 |  |
| Abnormal sperm parameters | 80(53.3) | 40(58.0) | 0.78(0.42~1.48) | 0.451 |
| **Infertility type** |  |  |  |  |
| Primary | 73(48.7) | 27(39.1) | 1 |  |
| Secondary | 77(51.3) | 42(60.9) | 0.63(0.32~1.24) | 0.181 |
| **Fertilization type** |  |  |  |  |
| IVF | 106(70.7) | 52(75.4) | 1 |  |
| ICSI | 44(29.3) | 17(24.6) | 1.38(0.64~2.94) | 0.412 |
| **Embryo transfer method** |  |  |  |  |
| Fresh | 92(61.3) | 40(58.0) | 1 |  |
| Frozen | 58(38.7) | 29(42.0) | 0.56(0.26~1.23) | 0.152 |
| **Type of embryos transferred** |  |  |  |  |
| Single cleavage-stage embryo | 5(3.5) | 3(4.3) | 1 |  |
| Double cleavage-stage embryo | 112(74.7) | 52(75.4) | 1.46(0.30~7.09) | 0.640 |
| Single blastocyst-stage embryo | 31(20.7) | 11(15.9) | 3.24(0.51~20.5) | 0.212 |
| Double blastocyst-stage embryo | 2(1.3) | 3(4.3) | 0.42(0.03~5.05) | 0.491 |
| **MTHFR C677T/A1298C** |  |  |  |  |
| CC/AA | 17(11.3) | 5(7.2) | 1 |  |
| CC/AC | 12(8.0) | 5(7.2) | 0.80(0.18~3.67) | 0.775 |
| CC/CC&CT/AA | 45(30.0) | 22(31.9) | 0.77(0.24~2.52) | 0.667 |
| CT/AC | 18(12.0) | 8(11.6) | 0.71(0.18~2.83) | 0.622 |
| TT/AA | 58(38.7) | 29(42.0) | 0.62(0.20~1.97) | 0.421 |

SD=standard deviation; aOR=adjusted odds ratio; BMI=body mass index; PCOS=polycystic ovarian syndrome; IVF=in-vitro fertilization; ICSI=intracytoplasmic sperm injection.

**Supplementary Table SX. Relationship between serum folate concentration (nmol/L) and combined *MTHFR* 677/1298 genotypes during folic acid supplementation in part of our study population (n = 103)**

| *MTHFR* genotype | adjusted(Mean±SEM) | Coefficient | *P* value |
| --- | --- | --- | --- |
| C677T/A1298C |  |  |  |
| CC/AA(n=11) | 36.80±2.40 | Ref. |  |
| CC/AC(n=10) | 33.53±2.47 | -3.27(-10.04~3.5) | 0.343 |
| CC/CC&CT/AA(n=33) | 30.86±1.36 | -5.94(-11.4~-0.48) | **0.033** |
| CT/AC(n=17) | 33.15±1.89 | -3.66(-9.64~2.33) | 0.231 |
| TT/AA(n=32) | 30.90±1.38 | -5.90(-11.35~-0.46) | **0.034** |

Generalized linear regression models were used to calculate the adjusted (Mean±SEM), coefficient, and P vale which were adjusted for age and BMI.

MTHFR=5,10-methylenetetrahydrofolate reductase; SEM: standard error of the means.

**Supplementary Table SXI. Relationship between serum homocysteine concentration (μmol/L) and combined *MTHFR* 677/1298 genotypes during folic acid supplementation in part of our study population (n = 217)**

| *MTHFR* genotype | adjusted(Mean±SEM) | Coefficient | *P* value |
| --- | --- | --- | --- |
| C677T/A1298C |  |  |  |
| CC/AA(n=21) | 6.31±0.71 | Ref. |  |
| CC/AC(n=20) | 6.22±0.73 | -0.10(-2.11~1.92) | 0.926 |
| CC/CC&CT/AA(n=82) | 6.86±0.36 | 0.55(-1.02~2.12) | 0.491 |
| CT/AC(n=31) | 6.10±0.58 | -0.21(-2.01~1.59) | 0.819 |
| TT/AA(n=63) | 6.89±0.41 | 0.58(-1.03~2.19) | 0.481 |

Generalized linear regression models were used to calculate the adjusted (Mean±SEM), coefficient, and P vale which were adjusted for age and BMI.

MTHFR=5,10-methylenetetrahydrofolate reductase; SEM: standard error of the means.
